# Prevalence of overweight and obesity among type 2 diabetic patients attending diabetes clinics in northern Tanzania

**DOI:** 10.1186/s13104-017-2861-9

**Published:** 2017-10-26

**Authors:** Damian J. Damian, Kelvin Kimaro, Godwin Mselle, Rose Kaaya, Isaac Lyaruu

**Affiliations:** 1 Kilimanjaro Christian Medical University College, P. O. Box 2240, Moshi, Tanzania; 2 Department of Community Medicine, Kilimanjaro Christian Medical Centre, P. O. Box 3010, Moshi, Tanzania; 3 Institute of Public Health, Kilimanjaro Christian Medical University College, Moshi, Tanzania

**Keywords:** Overweight, Obesity, Type 2 diabetes, Northern Tanzania

## Abstract

**Objectives:**

To determine the prevalence of overweight and obesity among patients with type 2 diabetes who are attending diabetes clinics in northern Tanzania.

**Results:**

In total 227 type 2 diabetic patients attending diabetes clinics were enrolled. Majority of patients 193 (85.0%) were overweight (44.9%) or obese (40.1%). Of them, 65 (33.7%) were overweight/obese after diagnosis of type 2 diabetes. The prevalence of overweight/obesity was significantly higher in female participants than the males [92.2% vs. 69.2%; OR = 5.10; 95% CI 2.22–11.05]. Regarding the region of residence, Kilimanjaro (100.0%) and Arusha (89.8%) regions had significantly highest prevalence of overweight/obesity compared to Tanga region (69.2%) [χ^2^ = 32.455, P < 0.001].

**Electronic supplementary material:**

The online version of this article (10.1186/s13104-017-2861-9) contains supplementary material, which is available to authorized users.

## Introduction

Non-communicable diseases (NCDs) are the leading cause of morbidity and mortality worldwide. In 2015, NCDs accounted for 70.1% of the global deaths [1]. Even in low and middle income countries like Tanzania, NCDs are now contributing to 34% of all deaths compared to 10% in the early 80s [1]. In 2014, World Health Organization (WHO) estimated 39 and 13% of adults (18 years and old) in the world being overweight and obese respectively [2]. Diabetes, the most common endocrine disorder, was estimated to affect 9% of adults worldwide [2]. Type 2 diabetes accounted for 90% of all diabetes cases globally [2].

A considerable amounts of literature have shown obesity to be a major modifiable risk factor for type 2 diabetes [3–6]. Moreover, overweight and obesity have been associated with profound health consequences including hypertension, hyperglycaemia, dyslipidaemia, coronary artery disease, cardiovascular diseases, cerebrovascular diseases, osteoarthritis, gallbladder diseases, respiratory tract diseases as well as psychological and emotional distress [7–9]. Overweight and obesity has further been linked with poor control of blood pressure, cholesterol and blood glucose levels among individuals with type 2 diabetes [10].

In Tanzania, there were estimated more than 822,800 cases of diabetes in 2015. The country recorded about 17,698 adult’s deaths due to diabetes in 2015 [11]. Despite high diabetes morbidity and mortality, the government has allocated funding for only diabetes treatment and prevention of secondary complications in public facilities. No funds for prevention and diagnosis are available with limited self-management education provided in the country [11]. The national diabetes and NCDs strategic plan has been developed since 2008. However, it has partially been implemented and currently in revision [11].

Weight loss among patients with type 2 diabetes has shown to provide beneficial impacts in treatment and control of metabolic parameters [12]. It is furthermore associated with a pronounced low cardiovascular risks among patients with type 2 diabetes [13]. Despite attending diabetic care, high proportion of type 2 diabetic individuals have been reported to be overweight or obese in different settings [14–17]. This increases risk of cardiovascular and microvascular diseases and mortality among type 2 diabetic individuals. In Tanzania, most of these patients after diagnosis are referred and attended in the districts, regional and tertiary care facilities whereby there is specialised diabetic care clinics. This is due to limited capacity of the primary care facilities in managing chronic diseases in the country. There is limited information on the prevalence of overweight and obese in people with type 2 diabetes who are attending specialized diabetic care clinics in northern Tanzania.

## Main text

### Study design and site

This was a facility based cross-sectional study conducted from February 2014 to July 2015 in three regions (Kilimanjaro, Arusha and Tanga) of the northern Tanzania. According to 2012 Tanzania population and housing census [18]; Kilimanjaro region has a population of 1,640,087, Arusha 1,694,310 and Tanga 2,045,205 people respectively. In these settings, facilities offering specialized diabetic care are Bombo hospital, Mt. Meru hospital and Kilimanjaro Christian Medical Centre (KCMC) in Tanga, Arusha and Kilimanjaro region respectively. KCMC is a referral hospital located in the slopes of snow-capped Mt. Kilimanjaro. It has been established by the Good Samaritan Foundation (GSF) since 1971 to serve people in the northern Tanzania. Bombo is a regional referral hospital located in Tanga municipal. It is a government facility serving people from within Tanga region. Mt. Meru is a regional referral hospital located in the foothills of Mt. Meru in Arusha municipality. It was established in 1926 and owned by the government to serve people from Arusha region.

### Study population and enrolment procedures

All patients with type 2 diabetes who attended outpatient diabetes clinics during study period were invited to participate. In this study, patients who were pregnant or lactating mothers were excluded. Patients meeting inclusion criteria and signed informed consent were enrolled. Data collection methods included face-to-face interviews with questionnaires and anthropometric measurements taking by the trained research assistants. Using closed-and open-ended questions, senior research scientists and medical student’s year 5 participated in the face-to-face interviews with type 2 diabetic patients. Questions on socio-demographic, economic, clinical and diagnostic characteristics were asked during the face-to-face interviews. Furthermore, information relating body mass index (BMI) at diagnosis were extracted from patient’s files.

### Measures

Anthropometric measurements (i.e. weight and height) were conducted following standardized procedures. One trained nurse and two medical students (year 5), conducted both anthropometric measurements in all regions. Body weight was measured when a patient was standing, wearing light clothes and without shoes using a digital scale (Seca, CA, USA). Height was measured when a patient was standing using a stadiometer. BMI was calculated as weight (kilograms) divided by height (metres) squared (kg/m^2^) and categorized as underweight (< 18.5 kg/m^2^), normal (18.5–24.9 kg/m^2^), overweight (25–29.9 kg/m^2^) and obese (≥ 30 kg/m^2^).

### Statistical analysis

Data entry, cleaning and analysis were done using Statistical Package for Social Science version 23.0 (SPSS, Chicago, IL, USA). Numerical data were summarized using measures of central tendency with their respective measures of dispersions. Frequency and percentages were used to summarize categorical data. *Chi square* test (χ^2^) was used to determine differences in prevalence of obesity and overweight by socio-demographic characteristics. Bivariate logistic regression analysis was used to determine patient’s characteristics predicting overweight/obesity among study population. Odds ratio (OR) with their corresponding 95% confidence intervals (CIs) were used to determine the strengths of association in a bivariate analysis. P value < 0.05 was considered statistically significant.

## Results

### Background characteristics of the participants

#### Socio-demographic and economic characteristics of respondents

A total of 227 type 2 diabetic patients attending specialised diabetes clinics in northern Tanzania were enrolled in this study, giving a response rate of 100%. The mean (± SD) age of respondents at enrolment was 56 (± 11.9) years. Slightly more than two-thirds of participants 154 (67.8%) were males. More than one-third of the patients (40.1%) were enrolled from Tanga; Kilimanjaro (33.9%) and Arusha (26.0%) region respectively. Most of the study participants were living in urban setting 141 (62.1%); with primary education 144 (63.4); engaged in unsalaried occupations 168 (74.0%) and unemployed 114 (50.2%). Table 1 shows the socio-demographic and economic characteristics of respondents.

**Table 1:** Socio-demographic and economic characteristics of respondents (n = 227)

Characteristics	n (%)
Age (years)	
≤ 40	20 (8.8)
41–60	129 (56.8)
61–80	74 (32.6)
81+	4 (1.8)
Mean ± SD	56 ± 11.9
Gender	
Male	73 (32.2)
Female	154 (67.8)
Region of residence	
Tanga	91 (40.1)
Kilimanjaro	77 (33.9)
Arusha	59 (26.0)
Setting of residence	
Urban	141 (62.1)
Rural	86 (37.9)
Level education	
No formal education	30 (13.2)
Primary education	144 (63.4)
Secondary education and above	53 (23.4)
Source of income	
None	19 (8.0)
Full time employment	28 (12.4)
Part-time employment	30 (13.3)
Self-fund retiree	16 (7.1)
Pension	22 (9.7)
Livestock keeping/agriculture	54 (23.9)
Dependent	58 (25.7)

#### Clinical and diagnostic characteristics of the study participants

The reported duration since lastly diagnosed with overweight/obesity ranged from 3 months to 33 years with median duration of 1 year. Most of respondents (62.9%) were diagnosed to be overweight/obese within 2 years prior recruitment to this study. The median reported duration since diagnosed with type 2 diabetes was 5 years ranging from 1 to 37 years. Almost one-third of the study participants (32.7%) were diagnosed with type 2 diabetes within 2 years prior to enrolment in this study. More than half of the study participants (57.2%) were overweight or obese at diagnosis of type 2 diabetes. Table 2 shows the clinical and diagnostic characteristics of the study participants.

**Table 2:** Clinical and diagnostic characteristics of the study participants (n = 227)

Characteristics	n (%)
Duration since diagnosed with overweight/obesity (n = 170) (years)	
≤ 2	107 (62.9)
3–5	25 (17.7)
6–10	28 (16.5)
11+	10 (5.9)
Median (range), years	1 (0.25–33)
Duration since diagnosed with type 2 diabetes (n = 153) (years)	
≤ 2	50 (32.7)
3–5	37 (24.2)
6–10	38 (24.8)
11+	28 (18.3)
Median (range), years	5 (1–37)
BMI at diagnosis of type 2 diabetes (kg/m^2^)	
< 18.5	7 (3.1)
18.5–24.9	27 (11.9)
25–29.9	63 (27.8)
≥ 30	64 (28.2)
Unknown	66 (29.0)

### Prevalence of overweight and obesity among study participants

In this study, the prevalence of overweight and obesity among type 2 diabetic patients attending diabetic clinics was 85.0% (n = 193). Of them, 44.9% were overweight and 40.1% were obese respectively. Additional file 1: Figure S1 depicts the prevalence of overweight and obesity among the study participants.

### Prevalence of overweight/obesity by socio-demographic characteristics

Table 3 shows the prevalence of overweight or obesity among type 2 diabetic patients attending specialized diabetic clinics by socio-demographic characteristics. Gender and region of residence were significantly associated with prevalence of overweight/obesity among study participants i.e. ($\chi^{2}$ = 19.417, P < 0.001) and ($\chi^{2}$ = 32.455, P < 0.001) respectively.

**Table 3:** Prevalence of overweight and obesity according to socio-demographic characteristics (n = 227)

Characteristics	Total N	Overweight/obesity N (%)		OR	95% CI
		No	Yes		
Age group (years)					
≤ 40	20	3 (15.0)	17 (85.0)	1.70	0.42–10.01
41–60	129	14 (10.9)	115 (89.1)	2.46	1.07–5.74
61+	78	18 (23.1)	60 (76.9)	1	
Gender (n = 227)					
Male	73	22 (30.1)	51 (69.9)	1	
Female	154	12 (7.8)	142 (92.2)	5.10	2.22–11.15
Region of residence					
Tanga	91	28 (30.8)	63 (69.2)	1	
Kilimanjaro	77	0 (0.0)	77 (100.0)	NC	
Arusha	59	6 (10.2)	53 (89.8)	3.93	1.44–12.39
Setting of residence					
Urban	141	19 (13.5)	122 (86.5)	1.36	0.59–3.01
Rural	86	15 (17.4)	71 (82.6)	1	
Level education					
No formal education	30	4 (13.3)	26 (86.7)	1.90	0.50–8.90
Primary education	144	19 (13.2)	125 (86.8)	1.93	0.78–4.59
Secondary	43	12 (22.7)	41 (77.3)	1	
Occupation					
Unemployed	115	24 (20.7)	92 (79.3)	1	
Employed	58	5 (8.6)	53 (91.4)	2.77	0.95–9.78
Self employed	54	6 (11.1)	48 (88.9)	2.09	0.76–6.64
Total	227	34 (15.0)	193 (85.0)		

*NC* not calculated

High prevalence of overweight or obesity were observed in female participants as compared to their male’s counterparts i.e. 92.2% vs. 69.9%. The results of bivariate logistic regression analysis showed females had fivefold increase in odds of being overweight/obese compared to males [OR = 5.10; 95% CI 2.22–11.15]. Regarding the place of residence, all patients residing in Kilimanjaro region were overweight or obese. Furthermore, high proportion of patients residing in Arusha region (89.8%) were overweight or obese as compared to those living in Tanga region (69.2%). Patients living in Arusha had almost 4 times higher odds of being overweight/obese compared to those living in Tanga [OR = 3.93; 95% CI 1.44–12.39].

Notably, the prevalence of overweight or obesity were marginally significantly different according to occupation and age of participants i.e. ($\chi^{2}$ = 5.313, P = 0.070) and ($\chi^{2}$ = 7.194, P = 0.062) respectively. Slightly vast majority of patients who were employed (91.4%) and self-employed (88.9%) were overweight or obese as compared to those who were unemployed (79.3%). Data shows being employed or self-employed had twofold increase in odds of being overweight/obese in this setting i.e. [OR = 2.77; 95% CI 0.95–9.78] and [OR = 2.09; 95% CI 0.76–6.64] respectively. Furthermore, the prevalence of overweight or obese was highest in patients aged 41–60 years (89.1%) and those ≤ 40 years (85.0%) comparing to those older than 60 years (76.9%). Patients ≤ 40 years had 70% higher odds of being overweight or obese compared to those older than 60 years [OR = 1.7; 95% CI 0.42–10.01]. Those aged 41–60 years, had twofold increase in odds of being overweight or obese compared to those who were older than 60 years [OR = 2.46; 95% CI 1.07–5.74].

However, the prevalence of overweight or obesity was not statistically significant according to the level of education and place of residence i.e. P = 0.250 and P = 0.417 respectively.

## Discussion

This study aimed to determine the prevalence of overweight and obesity among patients with type 2 diabetes who were attending specialised diabetic care clinics in Tanga, Arusha and Kilimanjaro regions, northern Tanzania. Generally, the study observed high prevalence (85.0%) of overweight/obesity among type 2 diabetic patients who were attending care clinics in these setting. This finding is in line with those reported from both population and facility-based studies conducted in Saudi Arabia [15, 19, 20], United States [21], United Kingdom [14], Nepal [22], Iran [23], Yemen [16] and Ghana [17]. High prevalence of overweight and obesity in this setting might be explained by the urbanisation, globalisation of food production and marketing, adoption of western culture and development of infrastructure that enhances little physical activities to our patients. Furthermore, limited policies on nutrition and regulations on marketing in the country might expose people unnecessarily to unhealthy feeding. Despite attending diabetic care clinic, there is limited self-management education offered by the health care workers mainly due to patient’s workload, staff shortage, and incomplete implementation of diabetes and NCDs strategic plans [11]. This might contribute to the observed high prevalence of overweight or obesity in this study population in our setting.

In this study we have observed a significant gender difference in the prevalence of overweight and obesity. There was high prevalence of overweight and obesity in females than males i.e. 92.2% vs. 69.9%. These results are in accord with earlier studies both prospective and retrospective which were conducted in both general population and facility-based among patients with type 2 diabetes [15, 19, 20, 24–28]. A possible reason for this difference might be physiological changes, genetic make-up, unhealthy eating behaviours and individual lifestyles among women. However, there might be other possible explanations for this difference.

Another important finding observed in this study was a significant difference in the prevalence of overweight and obesity by the place of residence. All patients enrolled in this study who were residing in Kilimanjaro region were overweight or obese. A possible explanation for these results may be enrolments of patients in the tertiary facility (KCMC). We hypothesize that, most of patients who attend care in a referral/tertiary facility (KCMC) to be in a critical condition or with poor prognosis.

At diagnosis, 56.0% of patients reported to be overweight or obese. The increase in proportion (29.0%) of patients who were overweight or obese in this study (change from diagnosis to enrolment) suggests lack of proper diabetic care and monitoring in the local setting.

## Limitation

The findings from this study might not be representative to all type 2 diabetes patients attending care clinics as we have not included those diagnosed from the primary care facilities but didn’t attend the specialised care clinics.

## Additional file

**Additional file 1: Figure S1.** The prevalence of overweight and obesity among study participants (n = 227).

## Abbreviations

BMI: body mass index
GSF: Good Samaritan Foundation
KCMC: Kilimanjaro Christian Medical Centre
KCMUCo: Kilimanjaro Christian Medical University College
NCDs: non-communicable diseases
RMOs: regional medical officers

## References

[1] World Health Organization. Global Health Estimates 2015: deaths by cause, age, sex, by country and by region, 2000–2015; 2016.http://www.who.int/healthinfo/global_burden_disease/estimates/en/index1.html. Accessed 22 Mar 2016.

[2] World Health Organization. Global Status Report on Noncommunicable Diseases 2014. Geneva. 2014. http://www.who.int/nmh/publications/ncd-status-report-2014/en/. Accessed 14 Dec 2016.

[3] Pinkney J (2002). Prevention and cure of type 2 diabetes. BMJ..

[4] UK Prospective Diabetes Study (1998). UK Prospective Diabetes Study. V. Characteristics of newly presenting type 2 diabetic patients: estimated insulin sensitivity and islet 4-cell function. Multi-centre study. Diabet Med.

[5] Abdullah A, Peeters A, de Courten M, Stoelwinder J (2010). The magnitude of association between overweight and obesity and the risk of diabetes: a meta-analysis of prospective cohort studies. Diabetes Res Clin Pract.

[6] Hossain P, Kawar B, El Nahas M (2007). Obesity and diabetes in the developing world–a growing challenge. N Engl J Med.

[7] Racette SB, Deusinger SS, Deusinger RH (2003). Obesity: overview of prevalence, etiology, and treatment. Phys Ther.

[8] Lyznicki JM, Young DC, Riggs JA, Davis RM (2001). Obesity: assessment and management in primary care. Am Fam Physician.

[9] Han TS, Feskens EJ, Lean ME, Seidell JC (1998). Associations of body composition with type 2 diabetes mellitus. Diabet Med.

[10] Anderson JW, Kendall CWC, Jenkins DJA (2003). Importance of weight management in type 2 diabetes: review with meta-analysis of clinical studies. J Am Coll Nutr.

[11] International Diabetes Federation. Global diabetes scorecard: tracking progress for action. http://www.idf.org/global-diabetes-scorecard (2014). Accessed 22 Mar 2016.

[12] Henry RR, Wallace P, Olefsky JM (1986). Effects of weight loss on mechanisms of hyperglycemia in obese non-insulin-dependent diabetes mellitus. Diabetes..

[13] Jung RT (1997). Obesity as a disease. Br Med Bull.

[14] Daousi C, Casson IF, Gill GV, MacFarlane IA, Wilding JPH, Pinkney JH (2006). Prevalence of obesity in type 2 diabetes in secondary care: association with cardiovascular risk factors. Postgrad Med J.

[15] Mugharbel KM, Al-Mansouri MA (2003). Prevalence of obesity among type 2 diabetic patients in Al-khobar primary health care centers. J Family Community Med.

[16] Al-sharafi BA, Gunaid AA (2014). Prevalence of obesity in patients with type 2 diabetes mellitus in Yemen. Int J Endocrinol Metab.

[17] Obirikorang Y, Obirikorang C, Anto EO (2014). Knowledge and lifestyle-associated prevalence of obesity among newly diagnosed type II diabetes mellitus patients attending diabetic clinic at Komfo Anokye Teaching Hospital, Kumasi, Ghana: a hospital-based cross-sectional study. J Clin Oncol.

[18] National Bureau of Statistics, Office of Chief Government Statistician. Tanzania Population and Housing Census 2012. Dar es Salaam, Tanzania. http://www.tzdpg.or.tz/fileadmin/documents/dpg_internal/dpg_working_groups_clusters/cluster_2/water/WSDP/Background_information/2012_Census_General_Report.pdf (2013). Accessed 22 Mar 2016.

[19] Abed Bakhotmah B (2013). Prevalence of obesity among type 2 diabetic patients: non-smokers housewives are the most affected in Jeddah, Saudi Arabia. Open J Endocr Metab Dis.

[20] Alqurashi K, Aljabri K, Bokhari S (2011). Prevalence of diabetes mellitus in a Saudi community. Ann Saudi Med.

[21] Hillier TA, Pedula KL (2001). Characteristics of an adult population with newly diagnosed type 2 diabetes: the relation of obesity and age of onset. Diabetes Care.

[22] Basukala A, Sharma M, Pandeya A. Prevalence of overweight and obesity among patients with type 2 diabetes mellitus in Kathmandu. Sky J o f Biochem stry Res. 2014;3(7):60–4. http://www.skyjournals.org/sjbr/pdf/2014pdf/Oct/Basukalaetalpdf.pdf. Accessed 22 Mar 2016.

[23] Marjani A (2011). Prevalence of obesity among type 2 diabetes mellitus in Gorgan (South East of Caspian Sea), Iran. J Chin Clin Med.

[24] Sibai AM, Hwalla N, Adra N, Rahal B (2003). Prevalence and covariates of obesity in Lebanon: findings from the first epidemiological study. Obes Res.

[25] Tur JA, Serra-Majem L, Romaguera D, Pons A (2005). Profile of overweight and obese people in a Mediterranean region. Obes Res.

[26] Njelekela MA, Negishi H, Nara Y (2002). Obesity and lipid profiles in middle aged men and women in Tanzania. East Afr Med J..

[27] El-Hazmi MA, Warsy AS (2000). Prevalence of overweight and obesity in diabetic and non-diabetic Saudis. East Mediterr Heal J.

[28] Arroyo P, Loria A, Fernández V (2000). Prevalence of pre-obesity and obesity in urban adult Mexicans in comparison with other large surveys. Obes Res.

