# Supplementary figures and images for: Prevalence of overweight and obesity among type 2 diabetic patients attending diabetes clinics in northern Tanzania
Source: BMC Res Notes. 2017 Oct 26;10:515. doi: 10.1186/s13104-017-2861-9 (PMC5659013; doi:10.1186/s13104-017-2861-9)

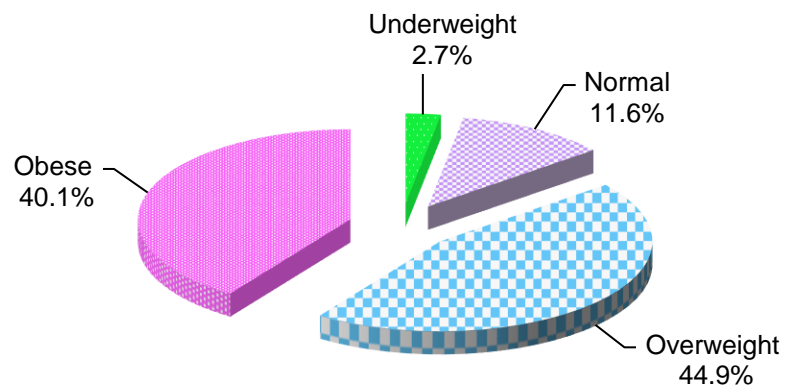

*Additional file 1: Figure S1. The prevalence of overweight and obesity among study participants (n = 227).*

Supplement: Supplementary file 1 — Additional file 1: Figure S1. The prevalence of overweight and obesity among study participants (n = 227). [file 13104_2017_2861_MOESM1_ESM.pdf]
